# Disruption in Surface-Based Functional Connectivity in the Right Posterior Hippocampal CA3 Subfield: A Probable Neural Basis of Visuospatial Working Memory Impairment in Patients With Right Temporal Lobe Epilepsy

**DOI:** 10.3389/fneur.2021.735689

**Published:** 2021-10-12

**Authors:** Zongxia Lv, Zirong Chen, Wei Ye, Xiaomin Pang, Liluo Nie, Weiwei Chang, Qijia Long, Jinou Zheng

**Affiliations:** ^1^Department of Neurology, First Affiliated Hospital of Guangxi Medical University, Nanning, China; ^2^Department of Radiology, First Affiliated Hospital of Guangxi Medical University, Nanning, China

**Keywords:** temporal lobe epilepsy, visuospatial working memory (VSWM), hippocampal subfield, surface-based functional connectivity (SBFC), default mode network subsystem

## Abstract

Visuospatial working memory (VSWM) impairment is common in patients with right temporal lobe epilepsy (rTLE). The posterior hippocampus is critical for spatial memory, but the contributions of the different subfields to VSWM deficits remain unclear. Forty-six rTLE patients and 42 healthy controls (HCs) were recruited. Resting-state fMRI (rsfMRI) and structural MRI scans were administered, followed by a VSWM_Nback test. The right posterior hippocampus was automatically segmented, and the surface-based functional connectivity (SBFC) of the subiculum (Sub), CA1, CA3, dentate gyrus (DG), hippocampal tail, and right entorhinal cortex (EC) were compared between groups. Correlation analysis was performed between the altered SBFC and VSWM_Nback scores for rTLE patients. The results showed that rTLE patients underperformed in the VSWM_Nback test, with longer mean reaction time of accurate response (ACCmeanRT) in 0back and 2back condition, lower hit rate (HR) and higher false alarm rate (FAR) in 2back condition. Compared with HCs, the rCA3 in the rTLE group exhibited decreased SBFC with inferior parietal cortex (IPC), temporal lateral cortex (TLC), and posterior visual cortex (PVC) in the right hemisphere as well as the bilateral dorsolateral prefrontal cortex (DLPFC). The SBFC of the rEC and right anterior cingulate cortex (rACC) increased in the rTLE group. Within the rTLE group, the decreased SBFC of the rCA3-rIPC and rCA3-rLTC were correlated with worse VSWM performance. Therefore, the decreased SBFC of the rCA3-rIPC and rCA3-rLTC might be the critical aberrant FC pattern reflecting VSWM impairment in rTLE patients. The mechanism might involve functional disruption between the core subsystem and the medial temporal subsystem of the default mode network (DMN).

## Introduction

Temporallobe epilepsy (TLE), the most common type in adult focal epilepsy, accounts for one-third of all refractory epilepsies. TLE patients can exhibit a range of cognitive impairments, among which working memory (WM) impairment is a common and important comorbidity (1). As the basis of higher cognition, WM deficits could adversely impact the quality of life of the patients (2). WM involves a series of information processes, including encoding, storage, manipulation, and retrieval (3). Depending on the different types of material being processed, WM can be categorized into two primary types, namely, verbal WM, and visuospatial WM (VSWM), of which the former activates more left-hemisphere areas, while the latter recruits more right-hemisphere areas (4). Many studies have assessed WM impairment in neurological and psychological diseases and have included research into the underlying mechanism of WM impairment in different diseases (5, 6).

Currently, the multi-component model of WM proposed by Baddeley and Hitch has gained widespread acceptance (3). The model contains four components that include the central executive machine, the phonological loop, the visuospatial sketchpad (VSS), and the episodic buffer (3, 7). VSS is an important element of VSWM; it involves the perception and maintenance of object and spatial location information and is associated with the function of the right dorsolateral prefrontal cortex (DLPFC), posterior parietal cortex (PPC), and occipital cortex (8). Neuroimaging evidence, especially information extracted from resting-state fMRI, has demonstrated that the right frontoparietal network highly overlaps with brain regions activated during the VSWM task (9). Also, we previously found that disruption of functional connectivity (FC) within this right lateralized network correlated with VSWM impairment in patients with right TLE (rTLE) (10). These findings suggest that the dysfunction within and between the right frontal and parietal cortices plays an important role in the pathophysiology of the VSWM deficit observed in rTLE patients.

Except for the role the cortical network played in WM, the hippocampus is the easiest to be remembered because it has a close relationship with memory. Patient H.M. is well-known to be barely able to generate new memories after receiving a bilateral medial temporal surgery, but still retains long-term memory 2 years before surgery, and his working memory has relatively remained (11). So, it has been a long time to associate hippocampus with long term-memory but not with WM. However, recently, several studies have reported the role of hippocampus involved in working memory performance (12, 13). It has been demonstrated that the hippocampus is greatly needed in tasks with complex high-resolution WM load (14). In addition, rodent studies have demonstrated that long-term potentiation (LTP) within the hippocampus was associated with the performance of spatial memory in the Morris water maze (15). Moreover, it has been shown that the oscillatory synchronization between the hippocampus and the prefrontal lobe is essential to maintain spatial WM (16).

Because of the vulnerability of the hippocampus to various pathological insults and deterioration of neurons due to aberrant discharges caused by recurring, long-term temporal seizures, numerous investigations have attempted to elucidate the relationship between WM impairment in TLE patients and hippocampal damage (1, 17). Winston et al. (17) reported the gray matter loss of ipsilateral hippocampus and parietal lobe in patients with right TLE and hippocampal sclerosis (TLE-HS) with WM deficit. Another study revealed that the greater decreased volume of the right hippocampus and parahippocampus in rTLE-HS patients correlated with a higher false detection rate in a spatial WM test (18). These evidences further emphasize the association between right hippocampus damage and VSWM impairment.

The improvements in neuroimaging techniques make it possible to analyze the subfields of the hippocampus in greater detail and gain new insights into hippocampal function. Currently, it is generally accepted that different hippocampal subfields exhibit specific anatomical and functional connectivity. During a WM process, the CA1 and subiculum (Sub) are involved in identifying the entire message from partial information (pattern completion), referring to retrieval and recall (19). Meanwhile, the CA3 and DG are responsible for separating similar items (pattern separation), primarily referring to encoding and maintenance (20). These are critical processes of WM for which any abnormality in the areas involved or their connections could result in WM loss. For instance, prior investigations have demonstrated that decreased right DG volumes correlate with worse spatial WM in school-aged children born preterm with very low birthweight (21). Electrophysiological evidence from rats suggested that modulating the spike ripple and the neuronal firing of CA3 could remarkably affect spatial WM and episodic memory (22). The Sub serves as a crucial gating region in the organization of hippocampal output and is primarily related to memory encoding and retrieval (19). In addition to estimating hippocampal subfields transversely, researchers also analyzed the hippocampus subregions in the longitudinal orientation, which revealed specific location–function relationships, including anterior–emotion and posterior–spatial memory and navigation (23). However, few current investigations have used the combined strategy of assessing vertical and horizontal levels to study the association between the FC of the hippocampal subfields in the posterior subregion and VSWM impairment in rTLE patients.

This study applied resting-state fMRI, the FreeSurfer 7.1.0 software, and DPABISurf_v1.6 to carry out a combined strategy (vertical and horizontal levels), focused on five right hippocampal subfields [e.g., rSub, rCA1, rCA3, rDG, and right hippocampal tail (rHT)] to analyze the FC of the regions of interest (ROIs) with the entire brain. An algorithm for surface-based FC (SBFC) was utilized to calculate the vertex-wise FC of the ROIs and compare the differences between rTLE patients and HCs. This algorithm presented an inflated original cerebral surface map of neuronal activity on the gyri and in the sulci. It also avoided disturbances from cerebrospinal fluid and white matter signals. Importantly, the algorithm reduced the false positives of activation around the boundary of adjacent but functionally separated brain regions, which probably occurred through the volume-based analysis (VBA) (24). Moreover, we analyzed the SBFC of the right entorhinal cortex (rEC), as this neighboring hippocampal formation is associated with spatial WM and makes considerable contributions to the transmission of information between neocortical areas and the hippocampus (25).

We hypothesized that the right posterior hippocampal subfields in rTLE patients might exhibit abnormal SBFC with critical cortical areas related to VSWM compared with HCs. Subsequently, for the rTLE group, the mean FCs were extracted from the regions that displayed significant differences during the intergroup comparison and were correlated with the neuropsychological VSWM_Nback scores. This was carried out to determine the central aberrant FC pattern that led to VSWM impairment and to uncover the possible compensatory mechanism, providing neuroimaging evidence for the early diagnosis, therapeutic target, and prognosis for rTLE patients with VSWM impairment.

## Materials and Methods

### Participants

Forty-six rTLE patients and 42 healthy adult participants matched for age, gender, and education were enrolled in this study (see Table 1). All rTLE patients were recruited from the Epilepsy Clinic of the First Affiliated Hospital of Guangxi Medical University. The rTLE patients were diagnosed by two experienced epilepsy specialists based on the diagnostic criteria outlined by the International League Against Epilepsy (ILAE) (26). All patients met at least two of the following inclusion criteria: (1) seizures with typical semiology suggesting an original epileptic focus in the temporal lobe; (2) brain MRI scans revealed atrophy or sclerosis of the right hippocampus, but no other specific abnormalities in the brain; and (3) ictal or interictal scalp electroencephalogram traces suggesting unilateral epileptic discharges in the right temporal lobe. The exclusion criteria included: (1) other kinds of systemic, neurological, or psychiatric diseases; (2) any MRI contraindications; and (3) any history of substance or alcohol abuse. All participants were right-handed and provided written informed consent. The study was approved by the Medical Ethics Committee for Clinical Research of Guangxi Medical University.

**Table 1:** Demographic and clinical data for right temporal lobe epilepsy (rTLE) and healthy controls (HCs).

	**HCs (*n* = 41)**	**rTLE (*n* = 45)**	***t/χ^2^***	***P-*value**
	**Mean (*SD*)**	**Mean (*SD*)**		
**Demographic characteristics**				
Gender (M/F)	16/25	16/29	0.11	0.74^a^
Age (years)	28.5 (7.7)	29.9 (8.9)	0.72	0.47^b^
Education (years)	15.1 (2.8)	14.2 (2.5)	−1.57	0.12^b^
**Clinical features**				
Age at onset (years)		19.6 (7.2)		
Epilepsy duration (years)		7.6 (4.6)		
Seizure frequency (times/month)		3.6 (2.8)		

*For all tests, p < 0.05 was considered statistically significant*.

a *P-Value was obtained by the chi-squared test*.

b *P-Values were obtained by two-sample t-test*.

*M, male; F, female; rTLE, right temporal lobe epilepsy; HCs, healthy controls; SD, standard derivative*.

### Magnetic Resonance Imaging Data Acquisition

Magnetic resonance imaging data were obtained using an Achieva 3T MRI scanner (Philips, Amsterdam, Netherlands) with a 12-channel head coil. High-resolution 3D structural T1-weighted images were acquired using a magnetization-prepared rapid gradient echo (MPRAGE) sequence with the following parameters: repetition time (TR) = 7.8 ms, echo time (TE) = 3.4 ms, flip angle (FA) = 9°, number of slices = 176, slice thickness = 1 mm, field of view (FOV) = 256 mm × 256 mm, and matrix = 256 × 256, resulting in an isotropic voxel size of 1 mm × 1 mm × 1 mm. Functional MRI data were acquired using an echo planar imaging sequence: TR = 2,000 ms; TE = 30 ms; FA = 90°; FOV = 220 mm × 220 mm; matrix = 64 × 64; in-plane resolution = 3.44 mm × 3.44 mm, slice thickness = 3.5 mm; interslice gap = 0.5 mm, and number of slices = 41, measuring a total of 225 volumes. To minimize head motion, foam padding was placed between the head and coil. To reduce scanner noise, each subject was given a set of earplugs. During scanning, the subjects were instructed to remain motionless, with their eyes closed, and not think about anything in particular. All subjects reported that they had not fallen asleep during the imaging protocol.

### Magnetic Resonance Imaging Data Analysis

#### Definition of Hippocampal Subfield Regions of Interest in Montreal Neurological Institute Space

**Figure 1:**
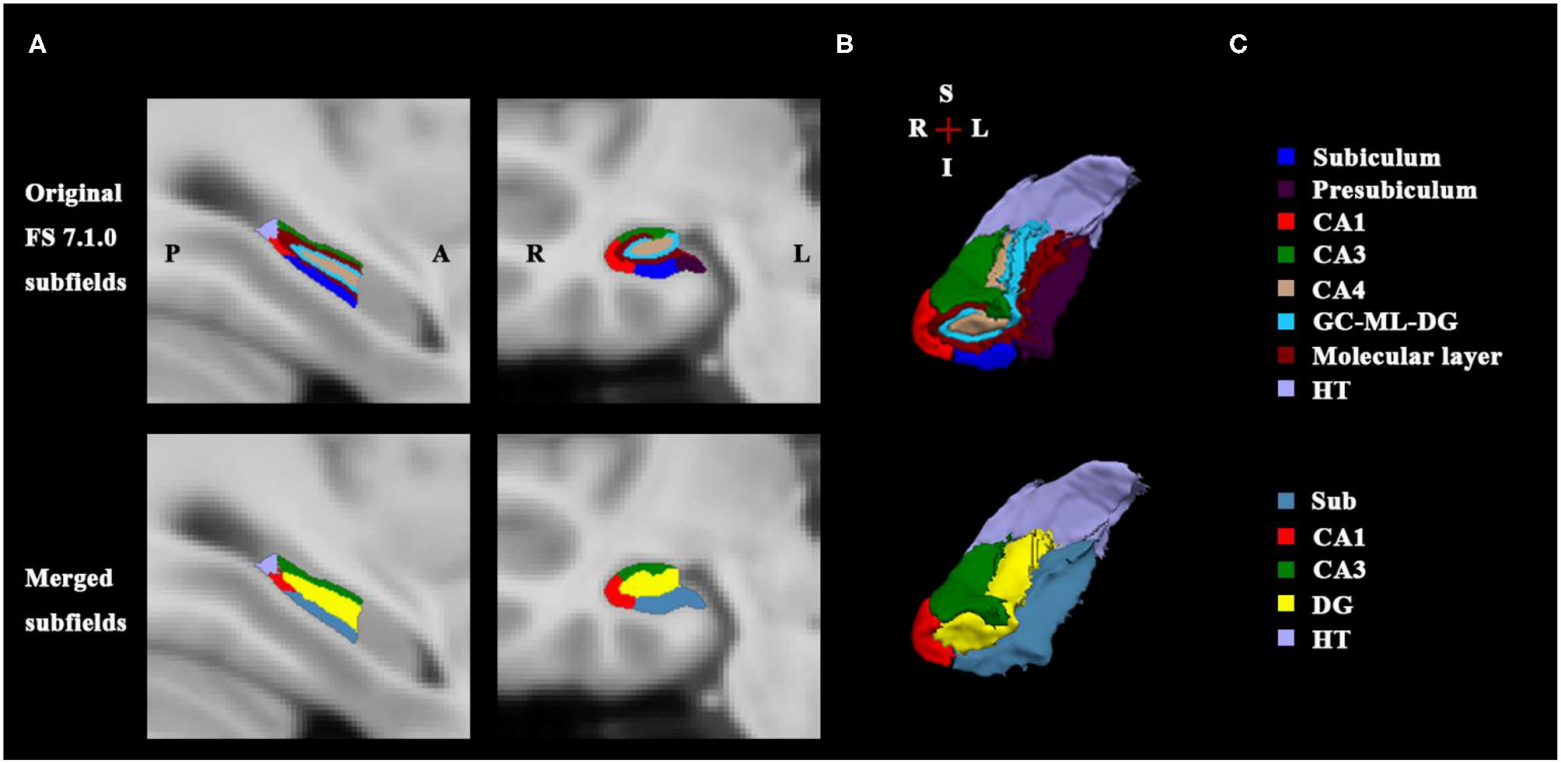
Right posterior hippocampal subfields of sectional view and 3D view. **(A)** Shows the sagittal and coronal slices of original FS 7.1.0 subfields (up) and the merged subfields (down). **(B)** Illustrates the 3D rendering of the hippocampal subfields generated from FS 7.1.0 segmentation (up) and from the merged method (down); The merged subfields are defined as the seed masks for the SBFC calculation. **(C)** Lists the corresponding subfield labels with specific color bar; the Sub includes FS labels subiculum and presubiculum; the DG comprises of FS labels CA4, GC-DG-ML, and molecular layer. FS, FreeSurfer; A, anterior; P, posterior; L, left; R, right; S, superior; I, inferior; HT, hippocampal tail; DG, dentate gyrus; CA, cornu ammonis; GC-ML-DG, granule cell layer and molecular layer of the dentate gyrus.

First, the structural template image of ICBM152_T1_1mm (27) in standard Montreal Neurological Institute (MNI) space was defined as the processing subject in the main recon-all pipeline of the FreeSurfer V7.1.0 software (http://surfer.nmr.mgh.harvard.edu), which is a fully automated image analysis package for brain structure segmentation. The technical details of these procedures have been described previously (28). Briefly, the process primarily includes motion correction, removal of non-brain tissue, automated Talairach transformation, segmentation of the subcortical white matter and gray matter (GM) structures, intensity normalization, tessellation of the white matter/GM boundary, automated topology correction and surface deformation, and alignment of the target image with an atlas constructed from a set of labeled training images. After these processes were completed, the gray matter structure of the hippocampus was extracted. Next, the hippocampal subfield segmentation protocol of the FreeSurfer was used to obtain different subfields in different subregions. This procedure is based on the Bayesian inference and a probabilistic statistical atlas of the hippocampal subregions built on ultra-high resolution, *ex vivo* MRI data from the autopsied brains (29). Sixteen hippocampal subfields were generated for each hemisphere, including CA1, CA3, CA4, the granule cell layer, and molecular layer of the dentate gyrus (GC-ML-DG), the molecular layer (ML), subiculum, and presubiculum within the hippocampal head and body subregions, respectively, as well as the parasubiculum in the body and the hippocampal tail in the posterior end. It was noted that CA4 is embedded into the backward-facing, flexed DG and includes a portion of the polymorphic layer of the DG. The ML lies between the CA regions and the GC-DG as well as the hippocampal fissure and consists of the molecular layer of the CA fields and subiculum. Therefore, we assessed a merged DG subfield in the body, in which the GC-DG-ML and CA4 were combined as one DG subfield, and the voxels in the ML were assigned the label of the closest voxel that was neither molecular layer nor background (29). The presubiculum and subiculum also were combined due to their common anatomical feature as the transition from the three-layered HC cortex to the six-layered cortex. Finally, for the right hippocampus, the CA1, CA3, DG, Sub in the body, and the hippocampal tail were ultimately selected as the FC seed ROIs in the MNI space and were resampled to the 2 mm isotropic voxel size (see Figure 1).

#### Anatomical and Resting-State Functional Data Preprocessing

The MRI data preprocessing was conducted using DPABISurf V1.6 (http://www.rfmri.org/DPABISurf) based on the Statistical Parametric Mapping software (SPM12, http://www.fil.ion.ucl.ac.uk/spm12) on the Matrix Laboratory platform (MATLAB R2018b, https://www.mathworks.com/). In general, the anatomical and resting-state functional data were preprocessed using the fMRIPrep 20.2.1 (30). Briefly, the procedure mainly included the following. First, the T1-weighted (T1w) image was corrected for intensity non-uniformity, and then the skull was stripped. Brain tissue segmentation for the cerebrospinal fluid (CSF), white matter, and GM was performed on the brain-extracted T1w data. Subsequently, the brain surfaces were reconstructed using recon-all (FreeSurfer). Volume-based spatial normalization to one standard space MNI152NLin2009cAsym (27) was performed through non-linear registration with antsRegistration (ANTs 2.2.0) (31), using brain-extracted versions of both T1w reference and the T1w template.

The preprocessing of the Blood Oxygen Level-Dependent (BOLD) functional data in the resting state was completed as follows. The initial five functional volumes were removed to achieve a signal equilibrium. Then the middle-time volume of each TR was defined as the reference volume, and its skull-stripped version was generated. Slice-time correction was conducted, and the BOLD reference was co-registered to the T1w reference with 6 degrees of freedom using bbregister (FreeSurfer), which implements boundary-based registration. After that, the head-motion parameters (transformation matrices and six corresponding rotation and translation parameters) were estimated. Subjects whose head motion exceeded 3 mm of displacement or 3° of angular rotation were excluded (Note that one rTLE patient and one HC were excluded for exceeding 3° of rotation). Normalization for surface data and volume data were performed in fMRIPrep. The processed BOLD series was normalized onto the FreeSurfer's fsaverage5 space with antsRegistration (ANTs). Meanwhile, the processed BOLD series was normalized into the MNI space and resampled into a 2-mm isotropic voxel size. Nuisance covariate regression was performed with the DPABISurf module for both surface and volume functional data. The Friston 24-parameter model (i.e., 6 head motion parameters, 6 head motion parameters one time point before, and the 12 corresponding squared items) was used to regress out head motion effects. Any participant with a mean frame displacement (FD) >2 mm was excluded. The white matter and CSF signals also were regressed out as covariates. We did not perform global signal regression (GSR) due to the controversy concerning its effect on the raw distribution of FC (32). Next, surface images were smoothed with a 6-mm full width at half maximum (FWHM) isotropic Gaussian kernel. Temporal filtering (0.01–0.1 Hz) was applied to reduce the effect of low-frequency drifts and high-frequency noise. As for the volume image, since the smooth processing may blur the signal contrast among the boundary of different subfields, the smooth procedure was not performed. After band pass filtering, the time courses of the right hippocampal subfields were extracted, respectively, and correlated with smoothed surface data during the calculation of the SBFC.

#### Calculation of Surface-Based Functional Connectivity

Given that two participants were excluded due to excessive head motion during fMRI scanning, 45 rTLE patients and 41 HCs were finally included in the subsequent SBFC calculation. The vertex-wise SBFC was calculated between the ROIs of the right hippocampal subfields and the brain surface regions in different hemispheres, respectively, followed by a Fisher-z transform. Then the intergroup differences were compared for the rTLE group vs. HCs. Additionally, given that the EC serves as a critical interface for interaction between the hippocampus and cortical regions and plays an essential role in spatial WM processing, the right EC surface region was selected and binarily masked as the FC seed ROI, using the image calculator utility implemented within DPABISurf. Then the vertex-wise SBFC of the rEC and brain cortical regions was calculated and compared with the between-group difference for rTLE vs. HCs.

### VSWM_Nback Neuropsychological Test

The VSWM_Nback test was adapted from the traditional N-back task described in our previous study (10). In brief, a letter “O” in white was randomly presented at one of the nine positions on a black background screen. The “O” was displayed in one position for 100 ms with an interleaved time of 1,000 ms before the next “O” was presented. The task included two conditions (0back and 2back). An instruction was displayed at the start of each condition for 4 s. In the 0back condition, each time the “O” was presented in the central position, a response button with the right index finger was required. In the 2back condition, each time the “O” was presented at the same position that it was in two presentations before, a button response was required. Each condition consisted of 15 stimuli, and five targets were randomly assigned. The two conditions were alternately switched 12 times, with a rest period of 30 s in the middle of the task. All participants were required to practice the entire test once and specifically the 2back condition twice to allow the subject to achieve a hit rate (HR) >75%. The HR (the number of accurate responses of the subject divided by the total number of accurate responses the subject should have responded) and the false alarm rate (FAR) (the number of error responses the subject actually made divided by the total number of error responses the subject should not have responded) as well as the mean reaction time of the accurate responses for 0back and 2back [ACCmeanRT (0back) and ACCmeanRT (2back)] were calculated. The participants were instructed to press the response button as quickly and accurately as possible and continue without considering whether they made a mistake.

### Statistical Analysis

Demographic data were compared using the SPSS 25.0 software (SPSS, Inc., Chicago, IL, USA). Continuous data were analyzed using two-sample *t*-tests, and categorical data were analyzed using chi-square tests. A *p* < 0.05 was considered statistically significant. VSWM_Nback scores were analyzed with two-way analysis of variance (ANOVA).

The neuroimaging statistical analyses were conducted using DPABISurf. The Z transformed SBFC map for each predefined right hippocampal subfield ROI was analyzed with a two-sample *t*-test to compare the rTLE group and HCs. The SBFC differences were analyzed using whole-brain analyses. Vertex *p* < 0.001 with cluster size *p* < 0.05 (*p* < 0.025 for each hemisphere, Monte Carlo simulation-corrected) was considered statistically significant. The abnormal SBFC results were visualized with DPABISurf. For the rTLE group, the mean z values for the SBFC in regions with significant alterations were extracted. Finally, Pearson's correlation was performed with multiple comparison correction between the mean *z*-values in regions with altered SBFC and the VSWM scores (HR, FAR, and ACCmeanRT), respectively, using SPSS 25.0 software.

## Results

### Demographic Assessment and Neuropsychological Test

The demographic data between the two groups were similar (see Table 1). The results of the VSWM_Nback test showed that the rTLE patients underperformed compared with the HCs, as shown by the two-way ANOVA results (see Table 2), there were significant main effects of group for reaction time (*F* = 46.29, *p* < 0.001), hit rate (*F* = 32.77, *p* < 0.001), and false alarm rate (*F* = 4.65, *p* < 0.001), and significant main effects of Nback for reaction time (*F* = 131.88, *p* < 0.001) and Hit Rate (*F* = 53.98, *p* < 0.001), the interaction of group × Nback only exists for hit rate (*F* = 26.54, *p* < 0.001). When compared with HCs, rTLE group exhibited longer reaction time of accuracy for the 0back and 2back conditions and higher false alarm rate for the 0back and 2back conditions. The simple effect analysis of group for hit rate showed that the rTLE exhibited lower hit rate for the 2back condition, but not for the 0back condition.

**Table 2:** VSWM_Nback performance between rTLE and HCs.

	**rTLE (*n* = 45)**	**HCs (*n* = 41)**		**Two-way ANOVA**		
	**Mean (*SD*)**	**Mean (*SD*)**		***F***	***p***	**η^2^**
**Reaction time (ms)**			Group	46.29	<0.001	0.221
0back	442.45 (68.23)	379.19 (42.56)	Nback	131.88	<0.001	0.453
2back	613.66 (101.48)	505.59 (89.05)	Group × Nback	2.53	0.11	0.025
**Hit rate (%)**			Group	32.77	<0.001	0.176
0back	99.77 (0.86)	99.57 (1.12)	Nback	53.98	<0.001	0.252
2back	83.32 (13.83)	96.75 (5.32)	Group × Nback	26.54	<0.001	0.142
**False alarm rate (%)**			Group	4.65	0.03	0.028
0back	2.48 (15.23)	0.13 (0.45)	Nback	1.29	0.26	0.008
2back	3.87 (4.45)	1.45 (1.58)	Group × Nback	0.02	0.89	0.000

*p < 0.05 was considered statistically significant. ANOVA, analysis of variance; rTLE, right temporal lobe epilepsy; HCs, healthy controls; SD, standard deviation*.

### Group Differences in Surface-Based Functional Connectivity Between the Right Temporal Lobe Epilepsy and Healthy Control Groups

**Figure 2:**
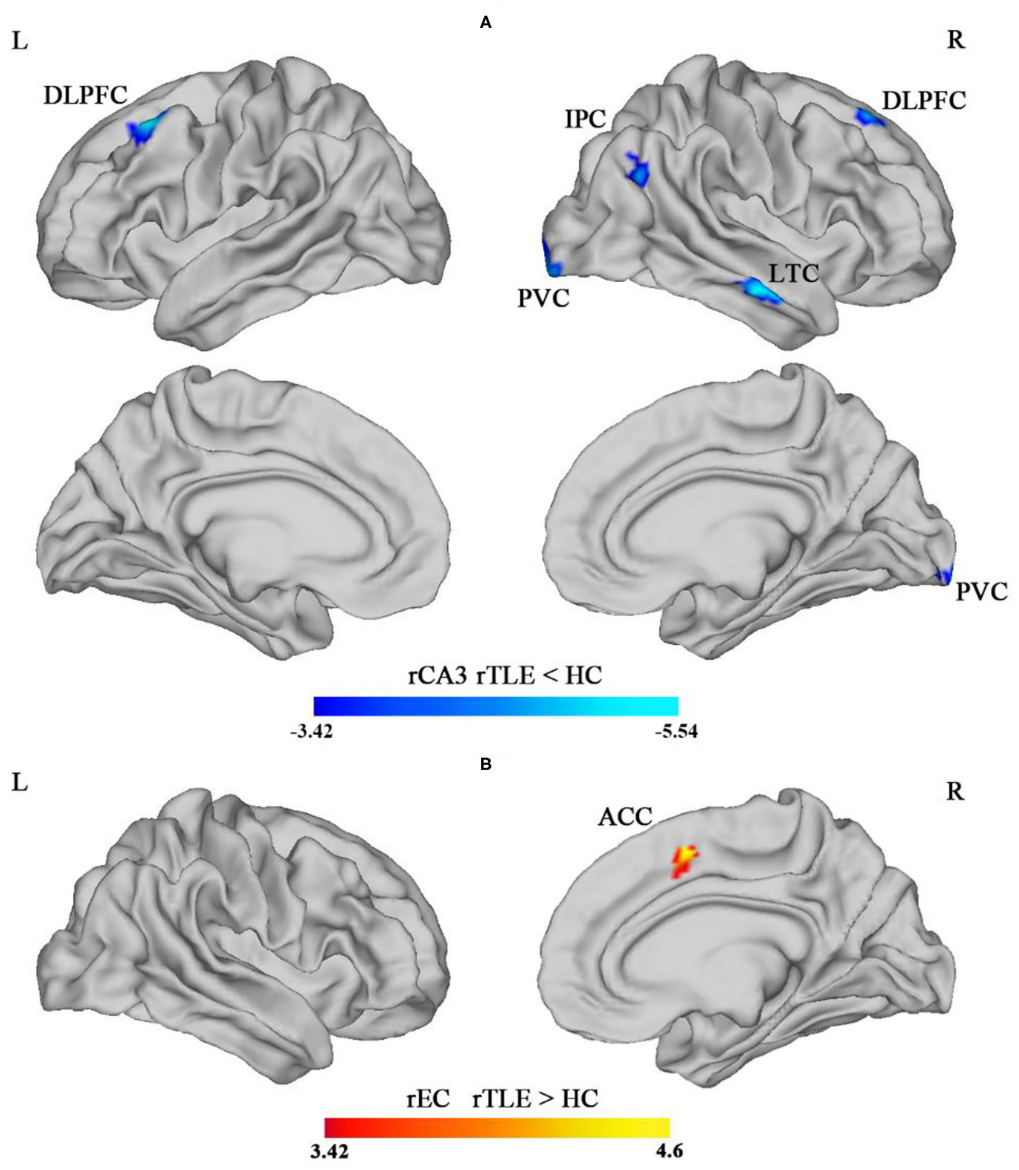
Intergroup difference of the surface-based functional connectivity (SBFC) of the right CA3 (rCA3) in the hippocampal body. **(A)** The blue-colored region illustrates the decreased SBFC with the rCA3 in bilateral dorsolateral prefrontal cortex (DLPFC), right lateral temporal cortex (LTC), right inferior parietal cortex (IPC), and right posterior visual cortex (PVC), comparing rTLE and HC (rTLE < HCs). **(B)** The red-colored region illustrated the increased SBFC of the right entorhinal cortex (rEC) with the right anterior cingulate cortex (ACC) (rTLE > HCs) [*p* < 0.05 (*p* < 0.025 for each hemisphere), corrected by Monte Carlo simulation].

The SBFC results revealed that, compared with the HC group, the rTLE group exhibited decreased SBFC with the rCA3 in cortical regions including the bilateral DLPFC, rIPC, rLTC, and rPVC (see Table 3; Figure 2A). On the other hand, the SBFC of the rEC and rACC was increased in the rTLE group (see Table 3; Figure 2B). No significant difference was observed for the SBFC of the rCA1, rDG, and right hippocampal tail in rTLE vs. HCs. All comparisons met the criterion in which the vertex *p*-value was <0.001, and the cluster size *p*-value was <0.05 (*p* < 0.025 for each hemisphere, Monte Carlo simulation-corrected).

**Table 3:** Intergroup differences of vertex-wise SBFC of the right posterior hippocampal subfields in rTLE vs. HCs.

**ROI**		**Region**		**HPC (vertices)**	**Cluster**	**Peak**	**Coordinates MNI**			***t***
					**Size (mm^**2**^)**	**Vertex**	***x***	***y***	***z***	
rCA3	rTLE < HC	L	DLPFC	67 (41) 68 (33) 73 (4) 97 (2)	808	218	−35	21	49	−5.54
		R	DLPFC	70 (13) 68 (5) 98 (1)	244	8,656	18	32	51	−4.45
			LTC	132 (18) 176 (9) 177 (8)	374	3,956	63	−17	−21	−5.01
			IPC	150 (34) 151 (6)	409	2,358	50	−63	27	−4.47
			PVC	1 (34) 4 (12)	558	10,007	21	−101	−12	−4.56
rEC	rTLE > HC	R	ACC	60 (21) 57 (14)	259	4,536	8	7	50	4.59

*Statistically significant differences in SBFC were defined as vertex p < 0.001 and cluster size p < 0.05 (p < 0.025 for each hemisphere), Monte Carlo simulation-corrected after correcting for age, sex, education, and head motion. MNI, Montreal Neurological Institute; HCP, Human Connectome Project; SBFC, surface-based functional connectivity; rTLE, right temporal lobe epilepsy; HCs, healthy controls; Sub, subiculum; DLPFC, dorsolateral prefrontal cortex; LTC, Lateral temporal cortex; IPC, inferior parietal cortex; PVC, posterior visual cortex; EC, entorhinal cortex; ACC, anterior cingulate cortex*.

### Correlation Analyses

**Figure 3:**
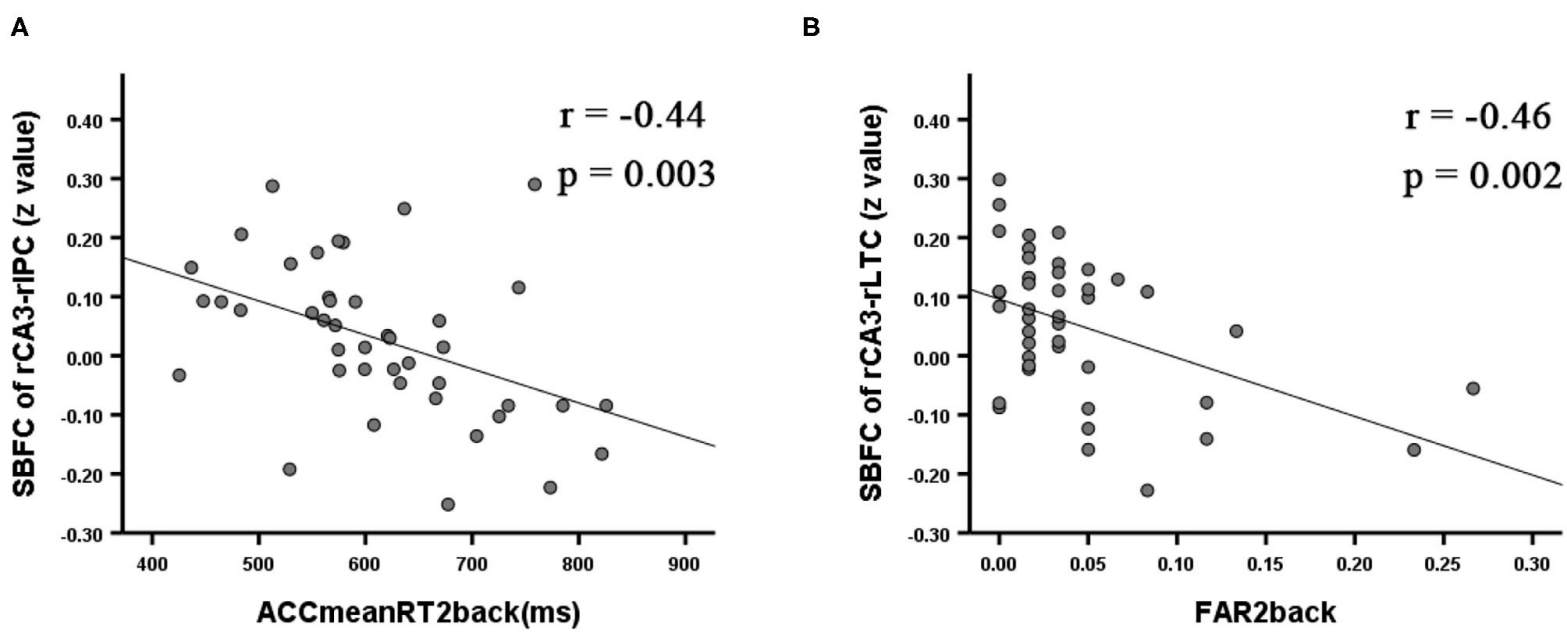
Significant correlation for the rTLE patients between the VSWM_Nback scores and the mean z value of the SBFC in regions with significant alterations during intergroup comparisons (*p* < 0.008 Bonferroni-corrected). ACCmeanRT2back was negatively correlated with the decreased SBFC of the rCA3-rIPC **(A)**. FAR2back was negatively correlated with the decreased SBFC of the rCA3-rLTC **(B)**. ACCmeanRT2back, mean reaction time for accurate responses in the 2back condition; FAR2back, false alarm rate for the 2back condition.

Correlation analyses between the VSWM_Nback test and the altered SBFC of the right posterior hippocampal subfield showed that, within the rTLE group, longer mean reaction time for accurate responses in the 2back condition was correlated with a weaker SBFC of the rCA3-rIPC (*r* = −0.44, *p* = 0.003; see Figure 3A). Higher false alarm rate for the 2back condition was correlated with a weaker SBFC of the rCA3-rLTC (*r* = −0.46, *p* = 0.002; see Figure 3B).

## Discussion

This substantial investigation analyzed the SBFC differences of the right posterior hippocampal subfields between rTLE patients and HCs and the impact on VSWM cognition. As for behavioral comparison, rTLE underperformed mainly in the 2back condition, implying a worse VSWM for information storing and manipulation. Notably, the higher false alarm rate for the 0back condition in the rTLE group might reflect the deficit in inhibitory executive function, which is important for WM. The present results revealed that the rTLE group exhibited cortical regions with decreased SBFC for the rCA3, including bilateral DLPFC, rIPC, rLTC, and rPVC, compared with the HC group. A prominent characteristic is that these aberrant regions were primarily distributed with the core subsystem of the default mode network (DMN) (33). Andrews-Hanna et al. (33) provided convergent evidence that the DMN could be subdivided into three subsystems, including the core subsystem, the medial temporal (MTL) subsystem, and the dorsal medial subsystem. Among these three subsystems, the core subsystem primarily involved self-reference, memory retrieval, rating, recollection, and judgment. These cognitive processes are highly correlated with VSWM. Moreover, a recent study has analyzed a resting fMRI data of healthy adults with data-driven method and demonstrated that the right posterior hippocampus belongs to a medial temporal network (MTN) (similar to the MTL-DMN defined by Andrews–Hanna) that engages in several processes that are critical for WM, including encoding, familiar perception, recognition, recall, and episodic retrieval (34). Also, the CA3 subfield, located at the top of the transverse section of the hippocampus, is a unique hippocampal subfield in that it contains numerous recurrent collaterals (also called auto-associative network), which are responsible for spatial associations and memory consolidation (20). Furthermore, the CA3 subfield participates in both pattern separation and pattern completion simultaneously and supports the temporary maintenance of spatial working memory (20). Collectively, it is reasonable to consider that the functional disconnection between these regions within two subsystems of the DMN could give rise to the disruption and inefficiency of essential processes involved in VSWM, which would manifest as worse VSWM performances in rTLE patients. This is consistent with the review of Ives-Deliperi and Butler (35) concerning about the resting state functional connectivity alteration in rTLE patients with VSWM deficit, in which they found significant decline of RSFC between medial temporal network and DMN, as well as within the frontal-insula parietal network.

The supposition described above was supported by the results of the correlation analysis in this study. Our findings showed that weaker SBFC of the rCA3-rLTC was correlated with longer mean reaction time for accurate responses in the 2back condition, indicating that the disruption of functional coordination and integration between the two regions might lead to a lower VSWM score. It has been documented that LTC is functionally and structurally correlated with the medial temporal cortex and hippocampus, which plays an important role in spatial memory (36). Notably, within the 2back condition, participants were required to accurately predict and recognize the spatial reappearance of the VSWM target stimuli. In other words, participants should have been able to judge rapidly whether the current stimulus appeared at the same spatial location as that of the stimulus two rounds before. It was possible that rTLE patients might falsely recognize the stimulus adjacent to the target stimulus as the correct choice due to dysfunctional spatial location discrimination caused by the pattern separation deficit in rCA3. Thus, the ACCmeanRT_2back was affected and presented as worse performances.

We found in the rTLE group that weaker SBFC of the rCA3-rIPC was correlated with higher false alarm rate for the 2back condition, suggesting that the dysfunction of right posterior hippocampus is related to VSWM impairment. This was supported by animal research, in which the dorsal hippocampus (dHPC) of rats were temporarily inactivated by infusions of the GABAa receptor agonist muscimol before a delay alteration task, and the results showed that the inactivation of dHPC increased the incidence of successive working memory errors (37). The rIPC is an essential cortical region for VSWM (38). It has a specialized function in storing visuospatial information as well as inner scribe and spatial movement rehearsal in VSWM through functional interaction with the rDLPFC (39). It is well-known that the executive control network (ECN), typically also called the task positive network (TPN), serves as a core component of Baddeley's multimodal WM system and takes charge of the functional coordination among different subsystems (7). Nevertheless, we noted that rCA3 and rIPC are regions within the DMN whose function is to exhibit deactivation during tasks and decorrelation with the ECN or TPN. Therefore, we proposed that the disconnection between these deactivated regions might reflect a disruption in coordination between the different DMN subsystems and further affect the degree of DMN deactivation during the task process, impairing the VSWM performance. This possibility is strongly supported by the research of Oyegbile et al., in which the level of DMN deactivation during the WM_2back condition was compared between pediatric TLE patients and HCs, with greater decreased DMN deactivation present in the TLE patients. The author considered this observation to be the result of a competition mechanism, by which the TLE patients could not easily diminish the participation of the DMN as well as recruit the ECN at the same time, resulting in worse WM performance (40).

In addition, the rIPC is an important area of the temporal–parietal junction (TPJ), which has distinct anatomical and functional connectivity with the hippocampus. Studies have demonstrated that the activation of the TPJ might reflect successful recollection, and abnormal FC of the TPJ with the hippocampus accompanied the underperforming WM (41). Collectively, we further proposed that the decreased FC of the rCA3-rIPC might be a central aberrant FC pattern that impairs VSWM performance in the rTLE patients. This inference is supported by a similar mechanism associated with schizophrenia. It postulates that hallucinations that occur in schizophrenia might be related to the abnormal FC of the TPJ with the hippocampus, which is usually observed in schizophrenia, and might result from damage to the hippocampus. For the schizophrenia patients, the hippocampal dysfunction might disable the inhibitory transmission from the hippocampus to cortical areas, causing excitation in the cortical regions, including the TPJ, visual regions, and auditory regions, and generating symptoms of hallucination (41). Similarly, in our study, the decreased FC of the rCA3-rIPC in rTLE patients might reflect the invalid suppression of the rCA3 to the rIPC, which could induce excitation of the rIPC and less deactivation of the DMN during the VSWM task, which would further affect the shift between the DMN and ECN, impairing the VSWM.

Our results showed that the SBFC of the right DLPFC with the rCA3 decreased in the rTLE group compared with HCs. It is well-known that the DLPFC is involved in information manipulation and operation during WM (42). This cluster is situated within the superior DLPFC and included 8Ad, s6-8, and 8BL, among which the 8Ad and s6-8 are WM-activated areas according to HCP MMP1 parcellation and meta-analysis (43). The decrease in SBFC might lead to a functional deficit in the manipulation of spatial information during VSWM. However, we did not find a significant correlation between the decreased FC and the VSWM scores. One reason for this observation is that the design of the VSWM_Nback test was not challenging enough because we did not include the 3back condition. It also is possible that the aberrant FC between the rCA3 and the posterior brain regions was more sensitive to VSWM impairment in the rTLE patients. We found SBFC was decreased between the rCA3 and the rPVC, which might suggest the disruption of the MTN in that the MTN was considered to serve as a bridge functionally connecting the visual cortex and the DMN/hippocampus (34). The impaired functional integration of rPVC, rCA3, and the MTN might impact the interaction between visual information and memory maintenance.

We also observed the increased SBFC of the rEC and the ipsilateral ACC regions, yet no significant correlation with VSWM performance. The ACC is the midline region of the salience network and refers to high-speed processing of the context and salient stimulus (44). Meanwhile, the EC is a crucial gating component of the information interaction between the hippocampus and neocortical regions, and the “grid cells” within the EC are engaged in encoding and updating spatial information (45). Neuronal tracer studies have verified that the EC receives afferent connections from the ACC, and might be related with memory (46). Additionally, the hippocampus also connects with the parietal lobe and frontal cortex, mediated by the ACC. It has been demonstrated that the bilateral frontoparietal network and the ACC are the main regions related to WM, and the fiber connections between them are the indispensable structural basis to make the network flow. The superior longitudinal fasciculus (SLF) and the cingulum are important fiber bundle, and the impaired white matter integrity has been reported to be associated with VSWM impairment (17). Given that the SLF connects DLPFC and parietal lobe, and projects to hippocampus through the cingulum, as well as the cingulum transforms information from ACC to EC, we proposed that the increased SBFC of rEC-rACC and the decreased SBFC of rCA3-rIPC and rCA3-DLPFC in this study might be a functional reorganization or adaptation.

Some limitations should be noted in this study. First, we cannot eliminate variations in the influence of different AED medications. Thus, designing a pair-wise longitudinal investigation would be meaningful. Second, the study was constrained by the relatively lower magnetic field of the fMRI. The accuracy of registration and functional spatial normalization of hippocampal subfields could be improved with the use of a 7-T fMRI and higher magnetic field conditions in the future. These conditions would make it possible to analyze the FC of the CA2, molecular layer, and granule layer of the DG. Third, we did not gather the information about HS histological type of rTLE patients. Given that the neurons in different subfields might be diversely damaged in different HS types and might induce distinct affection to memory, it should be considered in the future to retrospectively include rTLE patients with unified HS type according to the histopathological examination after anterior temporal lobectomy (ATL) or selective amygdalohippocampectomy (SAH) (47). In addition, we did not utilize more challenging conditions such as the VSWM_3back or dual tasks that would have required more complicated coordination within and between the subfields, which might have revealed more valuable information concerning the hippocampal subfields. Last but not least, we only focused on the whole brain SBFC of the right posterior hippocampal subfields. Future studies should be conducted that include the SBFC analysis of the hippocampal subfields and specialized networks such as the different DMN subsystems and right lateralized frontoparietal network. The functional metrics could be expanded to include effective or dynamic functional connectivity and graph theoretical analysis (48).

## Conclusion

Our results suggested that the decreased SBFC of the rCA3-rIPC and that of the rCA3-rLTC might be the critical aberrant FC pattern that reflects the VSWM impairment observed in rTLE patients. The mechanism might involve functional disruption between the core subsystem and the medial temporal subsystem of the DMN.

## Data Availability Statement

The raw data supporting the conclusions of this article will be made available by the authors, without undue reservation.

## Ethics Statement

The studies involving human participants were reviewed and approved by First Affiliated Hospital of Guangxi Medical University Ethics Committee. The patients/participants provided their written informed consent to participate in this study.

## Author Contributions

ZL: experimental design and writing—original draft. JZ: study concepts and funding acquisition. ZC: clinical assessment. WY and QL: MRI data acquisition. ZL and ZC: neuropsychological data collection. WC: data curation. ZL and XP: data analysis. LN and JZ: methodology. All authors contributed to manuscript preparation, read, and approved the final manuscript.

## Funding

This research was supported by the National Natural Science Foundation of China (contract authorization number: 81560223) and the Guangxi Postgraduate Education Innovation Plan (authorization number: YCBZ2019042).

## Conflict of Interest

The authors declare that the research was conducted in the absence of any commercial or financial relationships that could be construed as a potential conflict of interest.

## Publisher's Note

All claims expressed in this article are solely those of the authors and do not necessarily represent those of their affiliated organizations, or those of the publisher, the editors and the reviewers. Any product that may be evaluated in this article, or claim that may be made by its manufacturer, is not guaranteed or endorsed by the publisher.
